# HDL-C as a potential mediator between serum 25(OH)D and the angiographic severity of coronary artery disease: a single-center cross-sectional study

**DOI:** 10.1016/j.clinsp.2026.100996

**Published:** 2026-05-20

**Authors:** Marjan Mahdavi-Roshan, Zeinab Ghorbani, Morvarid Noormohammadi, Nargeskhatoon Shoaibinobarian, Sara Khoshdooz, Arsalan Salari

**Affiliations:** a Department of Clinical Nutrition, School of Medicine, Guilan University of Medical Sciences, Rasht, Iran; b Gastroenterology and Liver Diseases Research Center, Research Institute for Gastroenterology and Liver Diseases, Shahid Beheshti University of Medical Sciences, Tehran, Iran; c School of Medicine, Guilan University of Medical Sciences, Rasht, Iran

**Keywords:** Vitamin D, HDL-C, Gensini score, Mediation analysis, Dyslipidemia

## Abstract

**Introduction:**

Vitamin D deficiency has been associated with increased Coronary Artery Disease (CAD) risk, potentially through its associations with lipid metabolism, particularly HDL-C. This study investigates the mediating role of HDL-C in the relationship between serum vitamin D and the severity of CAD.

**Methods:**

In this cross-sectional study, 1015 participants undergoing elective angiography were enrolled from the Nutrition Heshmat Registry (NUTHER). Serum 25(OH)D levels were categorized as very low (< 12 ng/mL), low (12–20 ng/mL), marginal (20–30 ng/mL), and normal (≥ 30 ng/mL). CAD severity was quantified using the Gensini score, with severe CAD defined as a score ≥ 60. Logistic regression, restricted cubic spline analysis, and mediation models were employed to assess associations and potential mediation effects of HDL-C.

**Results:**

Participants with normal vitamin D levels (serum 25(OH)D ≥ 30 ng/mL) had 39% lower odds (adjusted OR = 0.61, 95% CI 0.40–0.93, P for trend = 0.017) of severe CAD compared to those with very low levels. A dose-response analysis confirmed a linear inverse association between 25(OH)D and CAD severity (P for overall = 0.0160). Mediation analysis revealed that HDL-C accounted for 18.1% (95% CI 11.8%, 44.0%) of the total effect of vitamin D on CAD severity, while LDL-C, total cholesterol, and triglycerides showed negligible mediation.

**Conclusion:**

Higher serum vitamin D levels are associated with lower CAD severity, partially mediated by HDL-C. These findings suggest that adequate levels of both vitamin D and HDL-C may be associated with a lower severity of CAD. Further research, including randomized trials, is needed to confirm these associations and explore clinical implications.

## Highlights

• Normal vitamin D lowers severe CAD risk 39%.
• Vitamin D shows inverse dose-response with CAD severity.
• HDL-C mediates 18% of vitamin D’s effect on CAD.
• LDL-C and triglycerides do not mediate vitamin D-CAD link.
• Adequate vitamin D and HDL-C may reduce CAD severity.

## Introduction

Coronary Artery Disease (CAD), a leading cause of mortality worldwide, has become a critical focus of global health efforts due to its association with the rising prevalence of non-communicable diseases.^1^^,^^2^ In 2021, Coronary Heart Disease (CHD) caused 375,476 deaths in the U.S., with annual heart attack cases averaging 605,000 new and 200,000 recurrent, and first occurrences at 65.6 years for males and 72.0 for females.^3^ CAD is closely associated with dyslipidemia and contributes significantly to the progression of atherosclerosis.^4^^,^^5^ Lifestyle interventions, particularly diet modification, are very important for the prevention of Cardiovascular Disorders (CVDs) by decreasing the risk of dyslipidemia.^6^

Dyslipidemia, a global condition characterized by increased total cholesterol, Low-Density Lipoprotein Cholesterol (LDL-C), and triglycerides, and decreased High-Density Lipoprotein Cholesterol (HDL-C), serves as a key contributor to CVD.^7^ Vitamin D is involved in various physiological functions, primarily calcium regulation and bone metabolism.^8^ Among nutritional deficiencies, vitamin D deficiency is the most prevalent, affecting nearly one billion people globally.^9^ Studies have shown that vitamin D deficiency (serum 25-hydroxyvitamin D (25(OH)D) <20‒30 ng/mL) is associated with a significantly increased risk of CAD,^10^, ^11^, ^12^ with one study presenting that serum 25(OH)D <30 ng/mL was associated with 5.8 times higher risk of CAD, independent of other risk factors.^10^

Previous research has demonstrated that lower vitamin D levels are negatively associated with LDL-C ^13^^,^^14^, or even positively associated with HDL-C.^13^, ^14^, ^15^, ^16^, ^17^Better lipid profiles, lower inflammation, and higher HDL-C levels are correlated with higher vitamin D levels, while the Monocyte/ HDL-C Ratio (MHR) serves as a marker for vitamin D deficiency 18, 19, 20 and a predictor of cardiometabolic diseases.^21^ In patients with chronic coronary syndrome, higher HDL-C and 25(OH)D levels have been observed, whereas ST-elevation myocardial infarction patients exhibit the highest MHR values. A significant correlation has been identified between 25(OH)D, HDL-C, and MHR, highlighting the interconnected roles of these markers in cardiovascular health.^19^ Vitamin D, primarily through its active form, calcitriol, is thought to influence lipid metabolism by binding to the Vitamin D Receptor (VDR), which may regulate bile acid synthesis and cholesterol production pathways. Vitamin D deficiency is correlated with disruptions in these processes and with dyslipidemia by promoting insulin resistance, lowering HDL-C levels, and increasing triglycerides and total cholesterol. This dysregulation is further exacerbated by increased intestinal absorption of fatty acids. Adequate vitamin D levels are also essential for suppressing Parathyroid Hormone (PTH), which helps lower triglyceride levels as well.^22^

Given the compelling findings from previous research that have revealed significant connections between serum 25(OH)D and HDL-C, it can be suggested that these factors may play a crucial role in cardiovascular health. This study aims to delve deeper into this relationship by examining the mediating effect of HDL-C on the link between serum vitamin D status and the severity of CAD, as quantified by the Gensini score. By investigating this pathway, our research aims to provide insights into potential biological mechanisms that may link vitamin D status, lipid profiles, and the severity of CAD.

## Method and materials

### Population and design

This single-center, cross-sectional investigation, conducted between January 2022 and June 2023 within the Nutrition Heshmat Registry (NUTHER) in Guilan Province, Iran, examined the relationship between serum 25(OH)D, HDL-C levels, and CAD severity assessed by the Gensini score. A total of 1235 adults aged 20‒80 years undergoing elective coronary angiography at Dr. Heshmat Hospital, Guilan University of Medical Sciences (GUMS), were enrolled after providing informed consent. Eligible participants were evaluated according to the “*ESC 2019 guidelines for the diagnosis and management of chronic coronary syndromes*”.^23^ Exclusion criteria included age < 20-years, BMI < 18.5 or > 40 kg/m^2^, history of CVD, and major chronic, metabolic, or inflammatory disorders.

The current study protocol within NUTHER was approved by the Institutional Review Board of the Cardiovascular Diseases Research Center, which is affiliated with GUMS (research no 1404020819), and the study was conducted in accordance with the 2013 guidelines of the Declaration of Helsinki. Furthermore, the investigation was authorized by the GUMS Ethics Committee (ethics code “IR.GUMS.REC.1404.089″). The objectives of the study were explained to the participants, and they provided their consent to participate through both oral and written communication. This study followed the Strengthening the Reporting of Observational Studies in Epidemiology (STROBE) guidelines for cross-sectional studies.

Further details on study design, recruitment procedures, and data collection have been described previously in related publications using the same NUTHER databank.^24^, ^25^, ^26^

### Demographic and anthropometric characteristics

On the admission day, participants were provided with a detailed explanation of the study's aims and objectives before signing informed consent forms to formally join the study. Demographic and socioeconomic data were collected by four skilled researchers during structured interviews. These included age, gender, employment and educational status, smoking habits, opiate use, and medical history. Patient medical records and prescriptions were reviewed to document chronic conditions and medication history. Data on medication use were collected, encompassing a wide range of therapeutic classes. These included antihypertensive agents, such as Angiotensin-Converting Enzyme (ACE) inhibitors, thiazide diuretics, calcium channel blockers, beta-blockers, and Angiotensin II Receptor Blockers (ARBs); antidiabetic medications, primarily metformin and sulfonylureas; lipid-lowering agents, mainly statins; anti-inflammatory drugs, including both corticosteroids and Nonsteroidal Anti-Inflammatory Drugs (NSAIDs); and anticoagulants such as clopidogrel, warfarin, enoxaparin, and rivaroxaban.

Anthropometric data, such as weight and height, were obtained using a Seca 755 medical scale with a precision of 0.5 kg. Heights were measured using a conventional stadiometer that was accurate to 0.1 cm. Measurements were conducted with the participants' shoulders in a neutral position and without their shoes. The BMI was determined as follows: (weight (in kilograms) / (height (in meters))^^2^).

Physical activity was assessed based on the valid questionnaire International Physical Activity Questionnaire (IPAQ), modified for the Iranian population.^27^

Dietary intake was assessed using a validated semi-quantitative Food Frequency Questionnaire (FFQ) administered by trained nutritionists. Daily energy intake (kcal/day) and macronutrient composition were derived using the Iranian Food Composition Table, supplemented with the USDA food database when necessary.^28^, ^29^, ^30^, ^31^

### Angiography

Upon admission, two cardiologists utilized the Judkin technique to perform coronary angiography using a femoral approach, thereby visually assessing the severity of atherosclerosis. The cardiologists, who were not informed of the study's specifications and serum 25(OH)D, interpreted routine angiograms. In a case of conflicting opinions, a third interventional cardiologist, who was also blinded to the study details, evaluated the angiograms to evaluate the degree. Details were described in the previous studies.^24^, ^25^, ^26^

### Gensini score calculation

The Gensini score was calculated using coronary angiography results based on the method described by Gensini et al.^32^ This scoring system assesses the CAD severity by evaluating the degree of stenosis in coronary lesions. The most significant stenotic lesions in each branch of the coronary arteries were quantitatively analyzed through angiography. Details were described in the previous study.^24^ The total Gensini score was determined by summing all the adjusted scores for their respective lesions.^33^, ^34^, ^35^

This study involved the classification of patients into two distinct groups according to their Gensini score: individuals with non-severe CAD (Gensini score < 60) and those with severe CAD (Gensini score ≥ 60). To further explore the current results, the authors also conducted sensitivity analysis based on additional cut-offs (Gensini scores of 50 and 70).

### Laboratory analyses

Participants fasted for a minimum of 8 h before providing 10 mL of venous blood. To prevent coagulation, the blood samples were preserved in tubes containing sodium citrate and stored at −20 °C until analysis. Leukocyte counts, including White Blood Cell (WBC) levels (10^9/L), neutrophil and lymphocyte percentages, and the Neutrophil-to-Lymphocyte Ratio (NLR), were determined using standard procedures. Fasting plasma glucose and total cholesterol concentrations were measured using enzymatic colorimetric methods, following the manufacturer's protocols.^36^ HDL-C was assessed using an enzymatic technique supplied by MAN Co. in Tehran, Iran. Triglyceride levels were determined by the glycerol phosphate oxidase enzymatic technique and commercial kits provided by Bionic Corporation (MAN Co., Tehran, Iran). LDL-C was determined using the Friedewald formula.^37^^,^^38^

Vitamin D levels in the form of serum 25(OH)D were evaluated using the Chemiluminescence Immunoassays (CLIA) method. Vitamin D levels were categorized according to serum 25(OH)D concentrations: very low levels as below 12 ng/mL, low levels as 12–20 ng/mL, marginal levels as 20–30 ng/mL, and normal levels as ≥30 ng/mL. These categories are based on the recommendations of the Endocrine Society and regional data on vitamin D deficiency^39^^,^^40^ with corresponding sample sizes of 169, 273, 284, and 289, respectively.

### Sample size calculation and statistical methods

Sample size estimation for the NUTHER study has been detailed elsewhere.^24^, ^25^, ^26^ In summary, based on a 95% confidence level (95% CI) and 0.03 precision, the minimum required sample was 926 participants. To allow for attrition and incomplete data, the target was increased by 25% to 1235. After excluding 35 participants with over 10% missing data, 1200 remained in the databank. Outliers were screened for dietary energy intake and biochemical measures; 185 participants with implausible energy intake values were excluded. The final analytical sample included 1015 participants.

Categorical variables were summarized using frequencies (percentages). Variations across serum 25(OH)D level categories were analyzed using Chi-Square or Fisher's exact tests. Regarding continuous variables, linear regression was employed to examine trends across serum 25(OH)D categories, with results presented as mean (Standard Deviations [SD]). To evaluate the likelihood of severe CAD, defined as a Gensini score ≥60, logistic regression analyses were performed using serum 25(OH)D both as categorical and continuous variables (per 10 ng/mL increase). The initial model included age and sex as covariates, while the fully adjusted models incorporated additional covariates, including BMI, smoking status, opiate use, employment status, medical histories related to hypertension, prediabetes, dyslipidemia, as well as medication usage encompassing antidiabetic drugs, anti-inflammatory agents, antihypertensives, antihyperlipidemics, and anticoagulants, alongside serum Neutrophil-to-Lymphocyte Ratio (NLR), daily energy intake (kcal/day), physical activity (MET min/day), and HDL-C levels (mg/dL).

Odds Ratios (OR) and 95% CI were then reported. Median for each category was treated as a continuous variable to facilitate the examination of linear trends (P-for-trend) across quartiles. Furthermore, to better investigate the robustness of the present findings on the association between 25(OH)D and odds of severe CAD, sensitivity analyses were run according to alternative cut-offs for Gensini score (i.e., ≥ 50 vs. < 50; and ≥ 70 vs. < 70).

Restricted Cubic Spline (RCS) regression with three knots at the 5th (8 ng/mL), 50th (22 ng/mL), and 90th (41 ng/mL) percentiles of serum 25(OH) D was applied to investigate potential nonlinear associations between serum 25(OH)D levels and the odds of severe CAD, with relevant covariate adjustments. The resulting data were visualized to depict estimated ORs and CIs from the spline model, illustrating the relationship between vitamin D status and the odds of severe CAD.

Subsequently, a mediation analysis was conducted to quantify the proportion of the total effect of 25(OH)D on severe CAD that is mediated through lipid parameters (HDL-C, LDL-C, total cholesterol, and triglycerides), using a product of coefficients approach with 1000 bootstrap replications to estimate confidence intervals.^41^, ^42^, ^43^ Serum 25(OH)D was specified as the independent variable, HDL-C, LDL-C, total cholesterol, and triglycerides as mediators, and the odds of severe CAD as the dependent variable. The total effect of serum 25(OH)D on severe CAD was decomposed into direct and indirect effects through lipid pathways. Interaction between serum 25(OH)D and HDL-C was evaluated by including a product term in a fully adjusted logistic regression model; the non-significant result (p-value = 0.110) justified the use of a standard mediation framework without interaction terms. All models were adjusted for age, gender, BMI, smoking status, opium use, employment status, hypertension, prediabetes/type 2 diabetes, dyslipidemia, medication use (antidiabetic, anti-inflammatory, antihyperlipidemic, antihypertensive, anticoagulant), NLR, total energy intake, physical activity, and HDL-C. The findings represent statistical decompositions and should be interpreted under the assumption of no unmeasured confounding among the exposure-outcome, mediator-outcome, and exposure-mediator relationships.

All statistical analyses were conducted using STATA version 17 software (StataCorp LLC, College Station, TX, USA).

## Results

Table 1 presents a comprehensive overview of participant characteristics in the current cross-sectional study, across different categories of serum 25(OH)D, with a total of 1015 subjects. The average age of participants was approximately 58 years, with no significant differences observed across the various serum 25(OH)D groups. The gender distribution was relatively balanced, with slightly over half of the participants being male. Significant differences were noted in employment types, marital status, and smoking prevalence, with the very low 25(OH)D group showing the highest rates of self-employment and farming (p-value = 0.024) and a greater prevalence of smokers (p-value = 0.014). In contrast, nearly all individuals in the marginal 25(OH)D category were married (p-value = 0.012). Analysis of past medical histories revealed significant disparities in hypertension prevalence (p-value = 0.018) and family history of CVDs (p-value = 0.010), with the marginal group exhibiting the highest rates for both factors. Additionally, biochemical and anthropometric evaluations indicated significant differences in HDL-C levels (p-value < 0.001), neutrophil and lymphocyte percentages (p-value = 0.001), NLR (p-value = 0.008), Gensini score (p-value < 0.001), and energy intake (p-value = 0.012). Participants in the higher serum 25(OH)D categories tended to have elevated HDL-C levels and lower NLR, Gensini score, and energy consumption (Table 1).

**Table 1:** Characteristics of the studied participants across categories of serum 25(OH)D, ng/mL) Levels in a cross-sectional study on Coronary Artery Disease (CAD) patients.

	Serum 25(OH)D concentrations categories				
Variables	Very Low	Low	Marginal	Normal	p-value^a^
	(< 12 ng/mL) (n = 169)	(12–20 ng/mL) (n = 273)	(20–30 ng/mL) (n = 284)	(≥ 30 ng/mL) (n = 289)	
Demographic data					
Age (y)	58.53 (10.81)	58.94 (9.99)	59.16 (11.07)	57.62 (10.61)	0.232
Gender, male, n (%)	101 (59.8%)	149 (54.6%)	145 (51.1%)	169 (58.5%)	0.204
Employment status					0.024
Self-employed, n (%)	83 (49.11%)	115 (42.12%)	108 (44.29%)	128 (42.76%)	
Employee, n (%)	28 (16.57%)	48 (17.58%)	62 (21.83%)	51 (17.65%)	
Housewife, n (%)	26 (15.38%)	66 (24.18%)	77 (27.11%)	79 (27.34%)	
Farmer, n (%)	32 (18.93%)	44 (16.12%)	37 (13.03%)	31 (10.73%)	
Married, n (%)	151 (89.3%)	244 (89.4%)	267 (94.0%)	248 (85.8%)	0.012
Smoking, n (%)	49 (29.0%)	46 (16.8%)	61 (21.5%)	52 (18.0%)	0.014
Opium, n (%)	34 (20.1%)	41 (15.0%)	52 (18.3%)	41 (14.2%)	0.278
Physical activity (min/day)	30.77 (27.46)	33.11 (27.41)	29.22 (26.91)	32.12 (28.32)	0.991
Past medical history					
Dyslipidemia, n (%)	145 (85.8%)	213 (78.0%)	234 (82.4%)	235 (81.3%)	0.219
Hypertension, n (%)	106 (62.7%)	196 (71.8%)	218 (76.8%)	207 (71.6%)	0.018
Prediabetes, n (%)	117 (69.2%)	186 (68.1%)	204 (71.8%)	191 (66.1%)	0.519
FHDM, n (%)	35 (20.7%)	84 (30.8%)	79 (27.8%)	85 (29.4%)	0.113
FHHTN, n (%)	46 (27.2%)	84 (30.8%)	90 (31.7%)	81 (28.0%)	0.673
FHMI, n (%)	31 (18.3%)	56 (20.5%)	52 (18.3%)	58 (20.1%)	0.878
FH cancer, n (%)	9 (5.3%)	29 (10.6%)	24 (8.5%)	26 (9.0%)	0.278
FHCVDs, n (%)	66 (39.1%)	129 (47.3%)	149 (52.5%)	118 (40.8%)	0.010
Medication use					
Anti-inflammatory drugs n (%)	135 (79.9%)	228 (83.5%)	225 (79.2%)	225 (77.9%)	0.377
Anticoagulant drugs, n (%)	15 (8.9%)	19 (7.0%)	31 (10.9%)	20 (6.9%)	0.267
Anti-hypertensive, n (%)	89 (52.7%)	153 (56.0%)	166 (58.5%)	164 (56.7%)	0.686
Anti-hyperlipidemic, n (%)	111 (65.7%)	151 (55.3%)	159 (56.0%)	169 (58.5%)	0.136
Antidiabetics, n (%)	74 (43.8%)	134 (49.1%)	151 (53.2%)	156 (54.0%)	0.140
Anthropometric, biochemical, and dietary data					
Body Mass Index (BMI) (kg/m²)	27.06 (4.44)	28.16 (4.79)	27.38 (4.29)	27.75 (4.50)	0.636
Fasting Blood Sugar (FBS) (mg/dL)	137.85 (75.33)	131.26 (58.68)	129.14 (49.43)	130.89 (65.76)	0.353
Cholesterol (mg/dL)	166.81 (53.34)	157.42 (44.72)	157.58 (43.48)	163.85 (47.47)	0.857
Triglycerides (mg/dL)	170.02 (104.94)	149.43 (79.61)	168.27 (116.31)	156.32 (84.87)	0.661
High-Density Lipoprotein (HDL-C) (mg/dL)	37.92 (9.11)	38.66 (9.61)	38.25 (9.34)	42.28 (13.21)	<0.001
Low-Density Lipoprotein (LDL-C) (mg/dL)	87.44 (31.89)	81.47 (29.23)	81.52 (28.84)	85.22 (30.86)	0.969
Neutrophil (%)	63.76 (11.27)	63.31 (10.13)	61.71 (9.20)	61.06 (9.67)	0.001
Lymphocyte (%)	30.91 (9.57)	31.90 (7.92)	32.55 (8.22)	33.47 (8.57)	0.001
Neutrophil-to-Lymphocyte Ratio (NLR)	2.52 (1.70)	2.21 (1.07)	2.23 (2.40)	2.05 (1.06)	0.008
Gensini Score	64.76 (36.29)	60.41 (40.02)	58.68 (39.78)	45.69 (38.64)	<0.001
Energy (kcal/day)	3313.88 (730.49)	3331.84 (712.94)	3347.64 (745.76)	3168.68 (722.90)	0.012

* All values are mean (SD), unless indicated.

a Linear regression for continuous variables and Chi-Squared test for categorical variables. FHDM, Family History of Diabetes; FHHTN, Family History of Hypertension; FHMI, Family History of Myocardial Infarction; FH cancer, Family History of Cancer; FHCVDs, Family History of Cardiovascular Diseases; LVEF, Left Ventricular Ejection Fraction.

The current study's findings, as presented in Table 2, highlight a significant association between serum 25(OH)D levels and the odds of severe CAD. Notably, individuals with normal vitamin D levels (serum 25(OH)D median = 37 ng/mL) had a substantially lower odds of severe CAD in contrast to those with very low levels (serum 25(OH)D median = 9 ng/mL). In the demographic-adjusted model (Model 1), participants with normal vitamin D levels (≥ 30 ng/mL) had a significantly lower odds of severe CAD (OR = 0.52, 95% CI 0.35‒0.76), compared to those with very low levels (< 12 ng/mL), with a statistically significant trend observed (P for trend = 0.001). This inverse association persisted even after adjusting for additional covariates, including BMI, smoking status, opium use, employment status, NLR, HDL-C levels, physical activity, total daily energy intake, as well as histories of chronic disorders and medications use individuals with normal 25(OH)D levels exhibited a 39% reduction in the odds of severe CAD (OR = 0.61, 95% CI 0.40‒0.93) for individuals with serum 25(OH)D levels ≥ 30 ng/mL (P for trend = 0.017). The robustness of this association was further confirmed by analyzing 25(OH)D as a continuous variable. Each 10 ng/mL increase in serum 25(OH)D was associated with a 18% lower odds of severe CAD in the demographic-adjusted model (OR = 0.82, 95% CI 0.74‒0.90), and an 14% lower odds in the fully adjusted model (OR = 0.86, 95% CI 0.77‒0.96).

**Table 2:** Odds Ratios (ORs) and 95% Confidence Interval (95% CI) of severe coronary artery disease (defined as Gensini score ≥60) according to serum 25(OH)D levels.

Serum 25(OH)D as categorical variable)					
Median Levels (ng/mL)	9	16	25	37	
Cases/non-cases	84/85	119/154	127/157	97/192	
Model^a^	1.00	0.79 (0.53, 1.16)	0.83 (0.57, 1.22)	0.52 (0.35, 0.76)	0.001
Model^b^	1.00	0.86 (0.57, 1.29)	0.84 (0.56, 1.27)	0.61 (0.40, 0.93)	0.017
**Per 10 ng/mL Serum 25(OH)D**					
Model^a^		0.82 (0.74, 0.90)			<0.001
Model^b^		0.86 (0.77, 0.96)			0.007

a Adjusted for age and gender.

b Additionally adjusted for Body Mass Index (BMI), smoking status, opium use, employment status, Neutrophil-to-Lymphocyte Ratio (NLR), total daily energy intake, physical activity, serum HDL-C levels, having a history of hypertension, prediabetes, and type 2 diabetes mellitus, or dyslipidemia, as well as use of antidiabetic, anti-inflammatory, antihyperlipidemic, antihypertensive, or anticoagulant medications.

**Fig. 1:**
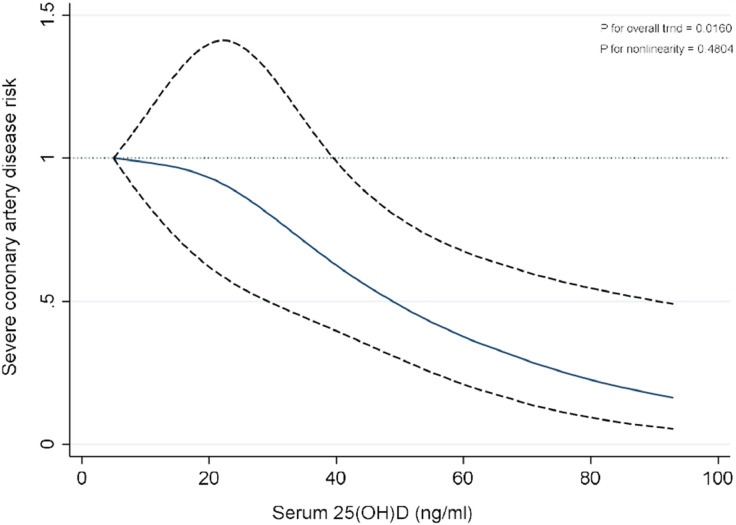
**A restricted cubic spline regression plot that depicts the relationship between serum 25(OH)D variability and severe Coronary Artery Disease (CAD) severity according to Gensini score** >**60.** The model accounts for factors such age, gender, Body Mass Index (BMI), smoking status, opium use, employment status, Neutrophil-to-Lymphocyte Ratio (NLR), HDl-C, total daily energy intake, physical activity, having a history of hypertension, prediabetes, and type 2 diabetes mellitus, or dyslipidemia, as well as use of antidiabetic, anti-inflammatory, antihyperlipidemic, antihypertensive, or anticoagulant medications. The spline model utilized cubic knots at the following percentiles of serum 25(OH)D: 5th (8 ng/mL), 50th (22 ng/mL), and 90th (41 ng/mL). The plotted lines represent Odds Ratios (ORs) and 95% Confidence Intervals (95% CIs).

Restricted cubic splines were used to assess and visualize the relationship between serum 25(OH)D variability and odds of severe CAD according to the Gensini score of >60. Fig. 1 shows the dose-response association after adjusting for all covariates. Meanwhile, the authors found a linear association between serum 25(OH)D and the odds of severe CAD (P for overall = 0.0160, P for nonlinearity = 0.4804), indicating that higher serum 25(OH)D concentrations were associated with lower odds of severe CAD (Fig. 1).

**Fig. 2:**
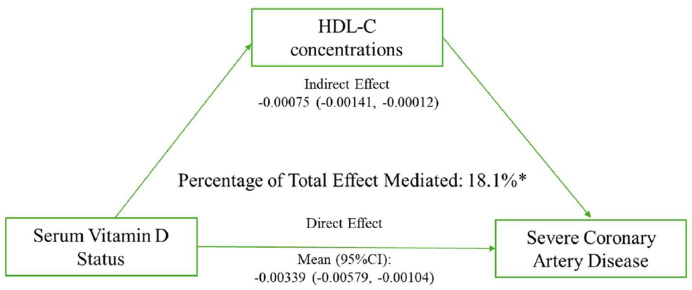
**Mediation analysis of the relationship between serum 25(OH)D and severe coronary artery disease odds by serum HDL-C concentrations.** * Indicates that approximately 18.1% of the effect of serum 25(OH)D on severe CAD appears to operate through the mediation of HDL-C. CI, confidence interval; HDL-C, high-density lipoprotein cholesterol.

A formal mediation analysis, adjusted for a comprehensive set of demographics, clinical, and lifestyle covariates, was conducted to quantify the extent to which the association between serum 25(OH)D and severe CAD (Gensini score ≥ 60) is mediated through specific lipid pathways. The results are summarized in Table 3. The analysis revealed that HDL-C levels served as a statistically significant mediator. For every 1 ng/mL increase in serum 25(OH)D, the Average Causal Mediation Effect (ACME) through HDL-C was −0.00075 (95% CI: −0.00141, −0.00012), indicating a significant indirect reduction in the log-odds of severe CAD. This mediation effect accounted for 18.1% (95% CI 11.8%‒44.0%) of the total effect of vitamin D on severe CAD. In contrast, the mediation effects through LDL-C, total cholesterol, and triglycerides were minimal and not statistically significant, as their 95% CIs for the ACME included zero. The proportions of the total effect mediated by these pathways were 1.3%, 0.9%, and 0.7%, respectively, confirming that HDL-C is the primary lipid mediator in this relationship (Table 3, Fig. 2).

**Table 3:** Mediation Analysis of the association between serum 25(OH)D (per 1 ng/mL increase) and severe coronary artery disease (gensini score ≥60) through lipid biomarkers.

Biomarker	Average Causal Mediation Effect (ACME)^a^ (95% CI)	Average Direct Effect (ADE)^a^ (95% CI)	Total Effect^a^ (95% CI)	Proportion Mediated (95% CI)
Serum HDL-C (mg/dL)	**−0.00075 (−0.00141, −0.00012)**	−0.00339 (−0.00579, −0.00104)	−0.00415 (−0.00639, −0.00171)	**18.1% (11.8%, 44.0%)**
Serum LDL-C (mg/dL)	−0.00005 (−0.00023, 0.00006)	−0.00393 (−0.00623, −0.00163)	−0.00398 (−0.00624, −0.00171)	1.3% (0.9%, 3.1%)
Serum Total Cholesterol (mg/dL)	−0.00004 (−0.00022, 0.00012)	−0.00394 (−0.00625, −0.00165)	−0.00398 (−0.00625, −0.00172)	0.9% (0.6%, 2.0%)
Serum Triglyceride (mg/dL)	−0.00003 (−0.00017, 0.00010)	−0.00394 (−0.00623, −0.00165)	−0.00397 (−0.00627, −0.00166)	0.7% (0.4%, 1.6%)

a All effects represent the change in log-odds of severe CAD for a 1 ng/mL increase in serum 25(OH)D. Models are adjusted for Body Mass Index (BMI), age, gender, smoking status, opium use, employment status, hypertension, prediabetes/type 2 diabetes, dyslipidemia, use of antidiabetic, anti-inflammatory, antihyperlipidemic, antihypertensive, or anticoagulant medications, Neutrophil-to-Lymphocyte Ratio (NLR), total daily energy intake, and physical activity. ACME and ADE estimates are based on 1000 bootstrap simulations. Bold indicates a statistically significant mediation effect (95% CI for ACME does not include zero).

To investigate the robustness of the present results, additional sensitivity analyses according to alternative cut-offs for Gensini score (≥ 50 vs. < 50; and ≥ 70 vs. < 70) were performed and yielded consistent results (Supplementary Tables S1‒S4). When defining severe CAD as a Gensini score ≥ 50, the inverse association persisted, with participants in the normal25(OH)D category having significantly lower odds in the fully adjusted model (OR = 0.45, 95% CI 0.29–0.68, P-for-trend < 0.001, Supplementary Table S1). Similarly, for a more stringent cut-off of Gensini score ≥ 70, the normal 25(OH)D category remained protective (OR = 0.63, 95% CI 0.41–0.96, P-for-trend = 0.010, Supplementary Table S2). The mediating role of HDL-C was also consistent across these sensitivity analyses, with the proportion mediated being 12.2% for the ≥ 50 cut-off and 26.1% for the ≥ 70 cut-off (Supplementary Tables S3 and S4).

## Discussion

Our study demonstrates an inverse association between serum 25(OH)D levels and the severity of CAD, as quantified by the Gensini score. After adjusting for potential confounders such as age, gender, BMI, smoking status, opium use, employment status, NLR, physical activity, total daily energy intake, HDL-C, and histories of chronic disorders and medication use, individuals with normal serum 25(OH)D levels exhibited significantly lower odds of severe CAD by about 39%, compared to those with vitamin D deficiency. Furthermore, a dose-response analysis revealed a linear relationship, with each 10 ng/mL increase in 25(OH)D associated with reduced odds of severe CAD.

To explore the underlying mechanisms, the authors conducted a formal statistical mediation analysis. The findings indicate that approximately 18% of the observed association between 25(OH)D and severe CAD is mediated by HDL-C levels. This proportion, while statistically significant, is modest in magnitude, suggesting that HDL-C is one of several pathways involved. It is important to note that our analysis measured HDL-C quantity, not its cardioprotective functionality, such as cholesterol efflux capacity or anti-inflammatory properties. It is plausible that vitamin D's more substantial impact lies in improving HDL-C function, a hypothesis that future studies with specialized assays should investigate. In contrast, other lipid biomarkers (LDL-C, total cholesterol, and triglycerides) demonstrated negligible mediation, underscoring the specific role of the HDL-C pathway. The robustness of these primary findings was confirmed in sensitivity analyses using alternative Gensini score cut-offs to define severe CAD.

These findings align with a body of evidence linking vitamin D deficiency to a higher prevalence and severity of CAD.^10^, ^11^, ^12^ For instance, one study reported that vitamin D deficiency was associated with a markedly increased odds of CAD, independent of traditional risk factors.^10^ Besides, the current evidence supports an increased odds of cardiovascular events in individuals with lower vitamin D levels. Among patients undergoing cardiovascular interventions, vitamin D deficiency is prevalent and linked to the severity of CAD.^44^, ^45^, ^46^, ^47^, ^48^

The proposed mechanisms underpinning this relationship are multifaceted. The specificity of HDL-C as a significant mediator, in contrast to the negligible roles of LDL-C, total cholesterol, and triglycerides, provides a compelling clue to the underlying biology. This pattern suggests that the link between vitamin D and CAD severity may operate less through the regulation of overall cholesterol burden or triglyceride-rich lipoproteins, and more through pathways where HDL-C plays a unique role. A plausible explanation lies in the shared anti-inflammatory and immunomodulatory properties of both vitamin D and functional HDL. Vitamin D is thought to be involved in cardiovascular health through potential mechanisms including the modulation of blood pressure, atherosclerosis, and lipid profiles. Specifically, vitamin D exerts anti-inflammatory and antioxidant actions, both of which are relevant to the pathogenesis of CAD. Vitamin D seems to effectively inhibit the intracellular NF-κB signaling pathway, thereby mitigating the progression of CAD.^49^, ^50^, ^51^, ^52^, ^53^, ^54^, ^55^, ^56^ Consequently, it downregulates pro-inflammatory cytokines like IL-6 and TNF-α and suppresses oxidative stress. The same inflammatory pathways are critically involved in rendering HDL-C dysfunctional, shifting it from a protective particle that promotes cholesterol efflux from macrophages to a pro-inflammatory state that loses its atheroprotective capacity. The reduction in inflammation and oxidative stress by vitamin D subsequently limits the formation of macrophage-derived foam cells, a crucial factor in the development of atherosclerosis.^49^, ^50^, ^51^, ^52^^,^^54^ Furthermore, vitamin D can modulate the expression of cholesterol efflux transporters such as ABCA1 and ABCG1 in macrophages, facilitating the efflux of cholesterol to HDL-C and limiting foam cell formation within atherosclerotic plaques by reducing LDL-C uptake by macrophages.^57^ This is supported by epidemiological data showing a positive correlation between 25(OH)D levels and HDL-C concentration. One study demonstrated that a 5 μg/L increase in 25(OH)D was associated with a 0.57 μmol/L rise in large HDL-C particles, independent of factors such as race, season, or total HDL-C concentration. While body fat partially influenced this relationship, age did not, underscoring the role of vitamin D in modulating lipid profiles and reducing cardiovascular risk factors.^58^ Another cross-sectional study found that serum HDL-C increased significantly with higher 25(OH)D levels, with a 4.2 mg/dL rise for every 10-ng/mL increase in serum 25(OH)D.^59^ Furthermore, vitamin D is associated with enhanced endothelial nitric oxide production and improved vascular reactivity, which may be related to the preservation of HDL-C function and integrity.^49^ However, the results of vitamin D supplementation trials on cardiovascular outcomes have been inconsistent,^49^ highlighting the complexity of this relationship and the need for further mechanistic investigation, as undertaken in our study.

Key strengths of this study include its relatively large sample size, the objective angiographic assessment of CAD severity using the Gensini score, and robust statistical adjustment for a comprehensive set of potential confounders. The use of mediation analysis adds a mechanistic dimension to our findings.

However, several important limitations must be emphasized. The most critical limitations include the observational, cross-sectional design, which prevents causal inference, and the lack of data on key determinants of vitamin D status, such as seasonal variation in blood draw and sunlight exposure. These unmeasured factors are potential sources of significant residual confounding. Other limitations include the absence of data on vitamin D supplementation, geographic latitude, Parathyroid Hormone (PTH) levels, Renal Function (eGFR), and detailed information on lipid-lowering therapy intensity. Finally, our single-center sample of patients undergoing angiography may limit the generalizability of our findings to broader, healthier populations.

### Future directions

Future research should aim to overcome these limitations. Longitudinal cohort studies with repeated measurements of 25(OH)D and seasonal adjustment are needed to establish temporality. To better assess causality, Mendelian randomization studies investigating genetically instrumented 25(OH)D and HDL-C traits could be highly informative. Ultimately, well-designed randomized controlled trials are warranted, which test vitamin D supplementation not just on clinical endpoints, but on intermediary functional endpoints such as coronary angiography, intravascular ultrasound, or measures of HDL-C function and vascular reactivity.

## Conclusion

In conclusion, this study provides evidence that sufficient serum 25(OH)D levels are associated with lower severe CAD. A statistically significant, though modest, portion of this association appears to operate through HDL-C levels. Given the high prevalence of vitamin D deficiency in Iran and similar regions, these findings may have relevant public health implications, suggesting that population-level strategies to improve vitamin D status could contribute to cardiovascular risk reduction. These insights highlight a potential interplay between vitamin D status and lipid metabolism in the context of cardiovascular health, justifying further investigation into the combined role of vitamin D and HDL-C, including its functional properties, in prospective and interventional studies.

## Ethical approval and consent

The study protocol was approved by the Institutional Review Board of the Cardiovascular Diseases Research Center, which is affiliated with GUMS (research no. 1404020819), and the study was conducted in accordance with the 2013 guidelines of the Declaration of Helsinki. Furthermore, the investigation was authorized by the GUMS Ethics Committee (ethics code “IR.GUMS.REC.1404.089”).

## Consent for publication

All authors have read and consented to the publication of this manuscript.

## Data availability statement

The datasets of the current study are available from the corresponding author on reasonable request.

## Funding

This study was financially supported by the Cardiovascular Diseases Research Center, GUMS (research code = 1403072309).

## CRediT authorship contribution statement

**Marjan Mahdavi-Roshan:** Conceptualization, Methodology, Supervision, Writing – review & editing. **Zeinab Ghorbani:** Conceptualization, Methodology, Formal analysis, Writing – original draft, Writing – review & editing. **Morvarid Noormohammadi:** Data curation, Formal analysis, Validation, Writing – review & editing. **Nargeskhatoon Shoaibinobarian:** Investigation, Resources, Writing – review & editing. **Sara Khoshdooz:** Investigation, Visualization, Writing – review & editing. **Arsalan Salari:** Data curation, Resources.

## Conflicts of interest

The authors declare no potential conflicts of interest concerning this article's research, authorship, and/or publication.

## Appendix Supplementary materials

Image, application 1
