# Supplementary material for: HDL-C as a potential mediator between serum 25(OH)D and the angiographic severity of coronary artery disease: a single-center cross-sectional study
Source: Clinics (Sao Paulo). 2026 May 20;81:100996. doi: 10.1016/j.clinsp.2026.100996 (PMC13213810; doi:10.1016/j.clinsp.2026.100996)
Supplement: Supplementary file 1 [file mmc1.docx]

**CLINICS-D-25-01442**

**Supplementary Materials**

| Table S1. Odds Ratios (ORs) and 95% Confidence Interval (95%CI) of severe coronary artery disease (defined as Gensini score ≥ 50) according to categories of 25-hydroxyvitamin D (25(OH)D). | | | | | |
| --- | --- | --- | --- | --- | --- |
|  | **Very Low**  **(< 12 ng/ml)**  **(n = 169)** | **Low**  **(12–20 ng/ml)**  **(n = 273)** | **Marginal**  **(20–30 ng/ml)**  **(n = 284)** | **Normal**  **(≥ 30 ng/ml)**  **(n = 289)** | **P for trend** |
| Median Levels (ng/ml) | 9 | 16 | 25 | 37 |  |
| ****Cases/Non-cases**** | 104 / 65 | 141 / 132 | 150 / 134 | 112 / 177 |  |
| ****Model a**** (Age, sex) | 1.00 (Reference) | 0.67 (0.45–1.00) | 0.71 (0.48–1.05) | **0.40 (0.27–0.59)** | **<0.001** |
| ****Model b**** (Full adj.) | 1.00 (Reference) | 0.75 (0.50–1.14) | 0.72 (0.47–1.09) | **0.45 (0.29–0.68)** | **<0.001** |
| ^a^ Adjusted for age and gender  ^b^ Additionally adjusted for body mass index (BMI), smoking status, opium use, work type, neutrophil-to-lymphocyte ratio (NLR), total daily energy intake, physical activity, HDL-C, having a history of hypertension, prediabetes, and type 2 diabetes mellitus, or dyslipidemia, as well as use of antidiabetic, anti-inflammatory, antihyperlipidemic, antihypertensive, or anticoagulant medications. | | | | | |

| Table S2. Odds Ratios (ORs) and 95% Confidence Interval (95%CI) of severe coronary artery disease (defined as Gensini score ≥ 70) according to categories of 25-hydroxyvitamin D (25(OH)D). | | | | | |
| --- | --- | --- | --- | --- | --- |
|  | **Very Low**  **(< 12 ng/ml)**  **(n = 169)** | **Low**  **(12–20 ng/ml)**  **(n = 273)** | **Marginal**  **(20–30 ng/ml)**  **(n = 284)** | **Normal**  **(≥ 30 ng/ml)**  **(n = 289)** | **P for trend** |
| Median Levels (ng/ml) | 9 | 16 | 25 | 37 |  |
| ****Cases/Non-cases**** | 65 / 104 | 99 / 174 | 105 / 179 | 75 / 214 |  |
| ****Model a**** (Age, sex) | 1.00 (Reference) | 0.92 (0.62–1.37) | 0.95 (0.64–1.41) | **0.57 (0.38–0.85)** | **0.004** |
| ****Model b**** (Full adj.) | 1.00 (Reference) | 1.03 (0.68–1.57) | 0.97 (0.64–1.47) | **0.63 (0.41–0.96)** | **0.010** |
| ^a^ Adjusted for age and gender  ^b^ Additionally adjusted for body mass index (BMI), smoking status, opium use, work type, neutrophil-to-lymphocyte ratio (NLR), total daily energy intake, physical activity, HDL-C, having a history of hypertension, prediabetes, and type 2 diabetes mellitus, or dyslipidemia, as well as use of antidiabetic, anti-inflammatory, antihyperlipidemic, antihypertensive, or anticoagulant medications. | | | | | |

| Table S3. Mediation Analysis of Serum 25-Hydroxyvitamin D (25(OH)D) Levels and Severe Coronary Artery Disease (CAD) (defined as Gensini score ≥ 50) odds according to the impact of Cholesterol Levels. | | |
| --- | --- | --- |
| Biomarker | **Proportion of total effect mediated**  **(mean (95%CI)** |  |
| Serum HDL-C (mg/dL) | 12.22% (8.67%, 23.47%) |  |
| Serum LDL-C (mg/dL) | 1.13% (-0.79%, 2.10%) |  |
| Serum Total Cholesterol (mg/dL) | 1.06% (-0.74%, 1.97%) |  |
| Serum triglyceride (mg/dL) | 0.10% (-0.07%, 0.18%) |  |

| Table S4. Mediation Analysis of Serum 25-Hydroxyvitamin D (25(OH)D) Levels and Severe Coronary Artery Disease (CAD) (defined as Gensini score ≥ 70) odds according to the impact of Cholesterol Levels. | | |
| --- | --- | --- |
| Biomarker | **Proportion of total effect mediated**  **(mean (95%CI)** |  |
| Serum HDL-C (mg/dL) | 26.07% (16.46%, 65.75%) |  |
| Serum LDL-C (mg/dL) | 2.57% (-1.59%, 6.64%) |  |
| Serum Total Cholesterol (mg/dL) | 1.27% (-0.78%, 3.21%) |  |
| Serum triglyceride (mg/dL) | 0.34% (-0.21%, 0.90%) |  |
